# A study on the mechanism of how network relationship strength affects the work development of college sports associations

**DOI:** 10.3389/fpsyg.2026.1772540

**Published:** 2026-04-20

**Authors:** Shuai Zhu, Anting She

**Affiliations:** Xinjiang University, Urumqi, China

**Keywords:** college, network ties strength, organizational performance, organizational ability, university sports association

## Abstract

College sports culture is rooted in campus environments, with college students as the main participants, sports activities as the core content, and campus spirit as the foundational support. As a key component of college sports culture, university sports association culture contributes significantly to enhancing students’ physical health and advancing the development of campus sports culture. To support students’ physical growth and the promotion of campus sports culture, it is essential to increase resource investment in campus sports initiatives, ensure the rational utilization of these resources, and thereby strengthen the operational capabilities of sports associations. However, university sports associations currently face notable operational challenges, primarily reflected in low organizational performance, difficulties in motivating students’ enthusiasm for sports participation, and consequent constraints on campus culture development. These issues stem from weak organizational capabilities of associations, a shortage of professional organizers, insufficient understanding of available association resources, and ineffective resource utilization. Fundamentally, the mechanism through which the network ties strength of university sports associations influences their organizational performance remains unclear, and empirical research exploring this relationship is lacking. Against this backdrop, this study focuses on investigating the influence mechanism of university sports associations’ network ties strength on organizational performance. This study first conducted a literature review and synthesis, then constructed an influence model to examine the relationship between network ties strength and organizational performance—with network ties strength as the independent variable, organizational ability as the mediating variable, and organizational performance as the dependent variable. Empirical research was carried out among members of sports associations in undergraduate colleges in Urumqi, using a combination of questionnaire surveys, field investigations, and expert interviews, resulting in the collection of 2,246 valid questionnaires. Correlation analysis, structural equation modeling, and regression analysis were employed to verify the research hypotheses. Finally, the study analyzed and summarized its findings, drew relevant conclusions, and put forward suggestions for improving the organizational performance of university sports associations. The results indicate that the influence mechanism of network ties strength on the organizational performance of university sports associations involves three core elements: network ties strength, organizational performance, and organizational ability. The specific mechanisms are as follows: (1) Both the network ties strength and organizational ability of university sports associations exert a positive impact on organizational performance; (2) the network ties strength of university sports associations has a positive influence on both organizational ability and organizational performance; (3) the organizational ability of university sports associations plays a partial mediating role in the relationship between network ties strength and organizational performance. This study clarifies the internal logical relationship between network ties strength and organizational performance of university sports associations, and provides theoretical support and practical guidance for optimizing the operation of university sports associations and promoting the high-quality development of campus sports culture.

## 1 Introduction

Globally, higher education institutions increasingly recognize university sports associations (USAs) as critical vehicles for advancing holistic student development—an objective aligned with UNESCO’s guidelines for quality physical education, which emphasize physical health, social integration, and lifelong exercise habits (Chandler, 1999). USAs operate at the intersection of formal physical education curricula and extracurricular engagement, enriching campus cultural diversity while enhancing students’ motor skills and physical fitness. Beyond these functional roles, USAs also foster the transmission of institutional sports spirit, merging athletic and humanistic values to shape students’ worldviews and support universities’ mission of promoting all-round development. Despite their significance, many USAs face persistent operational challenges, including low organizational performance, limited resource utilization, and inadequate student participation—issues that undermine their ability to fulfill these educational and cultural mandates.

A key underpinning of these challenges lies in the underleveraging of “network ties”—a concept from management and sociology referring to resources embedded in an organization’s relational networks. In corporate contexts, strong network ties have been consistently linked to enhanced organizational capability and improved performance. For resource-constrained campus organizations like USAs, such ties are particularly critical, yet their role in shaping USA performance remains underexplored. Existing research on USAs has predominantly focused on descriptive analyses of their cultural and educational functions or qualitative assessments of operational barriers, with limited empirical investigation into how network ties influence performance. Furthermore, while management studies have confirmed the positive impact of network tie strength on organizational outcomes, few have translated this framework to non-profit, campus-based settings. This gap is compounded by the lack of research examining mediating mechanisms—such as organizational ability—that might explain how network ties translate to improved USA performance. Corporate-derived models also cannot be directly applied to USAs, as the latter operate within unique institutional constraints that differ from for-profit entities.

To address these gaps, this study investigates the relationship between network tie strength (NTS), organizational ability (OA), and organizational performance (OP) in USAs, with a focus on undergraduate institutions in Urumqi, China. Urumqi serves as a representative context for northwest China’s higher education landscape: its universities with diverse disciplinary layouts and steadily improved educational resources reflect the development characteristics of regional higher education in western China, and the research findings on university sports associations here can provide empirical references for similar regional universities in China.

The primary objectives of this research are threefold: first, to develop a theoretical model that positions organizational ability as a mediator in the relationship between network tie strength and organizational performance of USAs; second, to empirically test this model using data from Urumqi’s undergraduate USAs, clarifying both direct and indirect effects of NTS on OP; and third, to derive evidence-based recommendations for enhancing USA performance through targeted improvements to network ties and organizational capabilities.

This study makes three key theoretical contributions (Anderson and Gerbing, 1988). First, it advances interdisciplinary scholarship by integrating network tie theory with sports science research, expanding the theoretical landscape of organizational performance in campus sports contexts. By applying Granovetter’s “Strength of Weak Ties”—a framework widely used in business but untested in USA research—it fills a critical gap in non-profit organizational studies. Second, it addresses methodological limitations in prior USA research, which has relied heavily on qualitative and theoretical approaches, by employing a quantitative design to provide rigorous empirical validation of theoretical claims. Third, it unpacks the “black box” of NTS-OP relationships by identifying organizational ability as a mediator, offering a more nuanced framework than linear, direct-effect models that have dominated existing research.

Practically, this study provides actionable guidance for USA administrators, university sports departments, and student leaders. Understanding which network ties most strongly predict performance can inform targeted resource investment. Insights into how organizational ability mediates NTS-OP relationships can also guide capacity-building initiatives, such as training for USA leaders in resource management and event planning. Given Urumqi’s leadership in Chinese higher education, recommendations derived from this study can serve as a blueprint for universities globally seeking to optimize their sports associations. Additionally, improved USA performance will directly benefit student well-being by increasing access to high-quality sports activities, thereby enhancing physical fitness and fostering lifelong exercise habits.

To achieve these objectives, a mixed-methods approach was adopted. A systematic literature review of English and Chinese sources informed the theoretical model and research hypotheses. Data were collected through a questionnaire survey of 2,246 valid respondents from USAs across Urumqi’s undergraduate universities, supplemented by field observations and semi-structured interviews with 5 experts and USA leaders. Statistical analyses, including descriptive statistics, reliability tests, and structural equation modeling (using SPSS 24.0 and AMOS 24.0), were employed to validate the proposed model. This multi-method design ensures both the empirical rigor and practical relevance of the study’s findings, contributing to both academic knowledge and the improvement of university sports associations worldwide.

This study makes three marginal contributions to the existing literature: First, it integrates network theory and organizational capability theory to construct a research framework for the performance influence mechanism of campus sports organizations, making up for the deficiency of single-theory application in existing research. Second, it takes university sports associations, a typical non-profit campus student organization, as the research object, expanding the application scenario of network relationship research from for-profit organizations and general non-profit organizations to campus sports organizations. Third, it empirically verifies the partial mediating role of organizational ability between network tie strength and organizational performance, revealing the internal transmission path of network relationship on the development of campus sports organizations, which is different from the direct-effect conclusion of most existing research.

## 2 Materials and methods

### 2.1 Research method

#### 2.1.1 Field survey method

A field survey was carried out on the sports clubs of undergraduate institutions in Urumqi, involving experts in the sports club research field, relevant person in charge, club participants, as well as the organization of club activities. The purpose of this survey was to gain an understanding of the daily operation of university sports clubs, the implementation of their activities, and the status of club participants, and to identify the shortcomings and problems existing in the current operation of sports clubs.

#### 2.1.2 Questionnaire survey method

Based on sports science knowledge and theories, design a questionnaire that conforms to the current development status of college sports associations. Select appropriate colleges and universities as samples for this study, with members of sports associations in undergraduate colleges in Urumqi as the survey respondents. Conduct questionnaire surveys and collect responses through both online “Wenjuanxing” (Questionnaire Star) and offline paper questionnaires to provide data support for this research.

#### 2.1.3 Method of mathematical collection and analysis

The data collected through the questionnaire survey method were processed, and valid questionnaires were screened out to serve as the basic database for this study. To address the potential common method bias caused by self-reported data, Harman’s single-factor test was adopted for ex-ante control in this study: all measurement items of the research variables were included in an unrotated exploratory factor analysis, and the results showed that the variance explained by the first common factor was 32.67%, which was lower than the critical value of 40%, indicating that there was no serious common method bias in this study. In addition, the questionnaire survey adopted anonymous filling and mixed the order of variable measurement items for ex-post control, so as to further reduce the impact of common method bias. The statistical software AMOS 24.0 and SPSS 24.0 were used to conduct relevant data processing and analysis on the valid questionnaires, including descriptive statistical analysis, reliability test, structural equation modeling and regression analysis, so as to test the theoretical hypotheses and research framework and thus obtain the statistical results of this study.

#### 2.1.4 Expert interview method

After analysing the data on sports associations in undergraduate colleges and universities in Urumqi obtained through the questionnaire survey method, the basic situation of sports associations at the current stage and the existing problems in their development will be identified. Based on these existing problems, interviews will be conducted with experts in the fields of physical education and management, as well as the presidents (or heads) of sports associations, with a total of 5 interviewees. The interview content includes the organization of sports association activities, the operation of the associations, and the research content of this thesis, aiming to obtain insights on the development of sports associations from them.

#### 2.1.5 System analysis method

Based on the review of literature, field surveys, data analysis results, and expert interviews, this study systematically summarizes and analyses the relationships and interactions among the network relationship intensity, organizational capabilities, and work performance of university sports associations in Urumqi at the current stage, and summarizes and refines suggestions from this research for the future development of university sports associations.

### 2.2 Literature review

The literature review in this chapter mainly describes three conceptual variables: the network relationship strength, work ability, and work performance of college sports associations. It mainly includes five parts: the first part defines the basic concepts of mechanism, college sports associations, network relationship strength, organizational ability, and organizational performance; the second part introduces the theoretical research on college sports associations; the third part introduces the theory of network relationship strength and previous research on it; the fourth part introduces the theory of organizational ability and previous research on it; the fifth part introduces the theory of work performance and previous research on it; the final part comments on the literature in the entire chapter.

#### 2.2.1 Literature review method

Papers were retrieved using keywords such as university sports associations, network ties strength, organizational ability, and work performance through the China National Knowledge Infrastructure database and the Xinjiang University Library system, which provided literature references for this thesis. By studying relevant contents about sports associations in existing management books and sports science, understanding their research methods, paths, and progress, and combining sports science theoretical knowledge to determine the research plan and put forward the hypotheses of the article, detailed data support was provided for this thesis.

#### 2.2.2 Definition and theoretical research of basic concepts

##### 2.2.2.1 Mechanism

Mechanism, originally referring to the structure and operational principles of a machine, is metaphorically used to denote the internal working mode of things, including the interrelationships among relevant components and the connections between various changes. It generally refers to the processes and methods of interaction between the organization or parts of a working system. It reflects the important role of organizational capability in the system. Therefore, the research variable of organizational capability is introduced to explore its significant impact.

##### 2.2.2.2 University sports associations

University sports associations refer to mass sports organizations within universities, which are spontaneously organized and voluntarily participated in by college students, with specific sports activities as the carrier.

Compared with foreign sports associations, China’s sports associations started relatively late, and research on them is relatively lagging behind. In terms of China’s current situation, there is a lot of research on associations in general, but in the early stages of development, there was less research on sports associations, and most of it focused on national or regional sports associations. Research on college students’ sports associations is even less, which is clearly reflected in the number of articles retrieved from CNKI.

However, at present, as college sports associations are playing an increasingly important role in the sports cause of college students in China, the majority of sports workers and students majoring in related fields are paying more and more attention to the research on college sports associations. I have reviewed over 1,000 publicly published journal articles, doctoral dissertations, and master’s theses from 2010 to 2018 related to “college sports associations”. The number of studies showed a fluctuating upward trend starting from 2010, reaching a peak in 2016 before gradually declining. For details, please refer to Table 1.

**Table 1:** Statistics on the number of scientific research papers related to college sports associations from 2016 to 2024.

Year	Number of CNKI documents
2016	225
2017	260
2018	253
2019	321
2020	338
2021	389
2022	422
2023	325
2024	268

##### 2.2.2.3 Network ties strength

Network relationship strength, as an important characteristic variable in network relationship research, is used to indicate the strength of the relationship between two nodes in a network. In the field of mathematics, a network is a type of graph (Barden, 2007), and it generally refers specifically to a weighted graph. In the field of computers, a network is a virtual platform for information transmission, reception, and sharing. It connects information from various points, surfaces, and entities, thereby enabling the sharing of these resources. Network relationship strength is an important indicator reflecting the characteristics of network relationships. The network relationship strength of college sports associations studied in this paper mainly refers to the degree of closeness in the connections between sports associations and other behavioral subjects, including association members, other associations in the same school, sports associations in other colleges and universities, sponsoring organizations, and other related organizations.

Johns and Demarche were the first to use the term “strength” to compare the power of connections in interactions between organizations. Granovetter first introduced “Strength” into network theory in his published article “The Strength of Weak Ties”. He pointed out that there are differences in strength in the communication and influence between people, between people and organizations, and between organizations. Based on the differences in the strength of network relationships, the relationships between the two parties are divided into Strong Tie and Weak Tie (Burt, 1992). Since then, the strength of network relationships has always been a focus concept in the research of sociology and management.

There is relatively little research on the strength of network relationships in sports science. Since relationship strength consists of abstract concepts such as intimacy that are difficult to specifically quantify, there is still no unified conclusion in academia on the definition of online relationship strength at this stage (Baron and Kenny, 1986), and the measurement of relationship strength is also in a relatively weak link. Granovetter (1973) argued that the strength of a relationship should include four aspects: the communication time between nodes, the closeness of emotions, familiarity, and reciprocity. In subsequent studies, scholars have also attempted to expand the measurement of network relationship strength from different perspectives. The indicators for measuring the strength of network relationships in their research are shown in Table 2.

**Table 2:** Measurement indicators of network relationship strength.

Measure	Author
Tightness, sociability	Yanjie Bia (2020)
Frequency of communication, degree of intimacy, degree of mutual trust, duration of the relationship	Nelson (2016)
The frequency of interaction, the degree of intimacy	Levin (2024)
Reciprocity, trust, intimacy, giving or receiving advice	Mathews (2018)
Sociability, intimacy, frequency	Mitchell (2019)
Frequency, the intensity of emotions, the scope of common topics, intimacy	Perlman and Fehr (2021)

Studies related to the strength of network relationships are usually associated with other variables, and these variables interact with each other, resulting in different paths of relational influence. After sorting out and summarizing a large number of literatures, it is found that research on the strength of network relationships mainly takes it as a dependent variable to study its influence and influence paths on other variables. The research can be divided into two categories: one is to study the strength of network relationships as a whole, and the other is to subdivide network relationships into strong network relationships and weak network relationships for research.

##### 2.2.2.4 Organizational capability

Organizational capability refers to the ability to carry out organizational work. It includes a set of capabilities possessed by an organization that reflect efficiency and effectiveness. There is no unified conclusion on the constituent dimensions of organizational capabilities. Among them, the more representative studies are put forward by Eisenhardt, Gold, and Teece. Eisenhardt (2000) summarized organizational capabilities into five dimensions in his research: excellent assets, cognitive elements, procedures and routines, organizational structure, and behavior and culture; Gold (2001) believed that organizational capabilities include two dimensions: organizational efficiency and organizational members’ satisfaction (Blumstein and Kollock, 1988); Teece (1997) pointed out in his article that organizational capabilities should be studied from the dimensions of organizational effectiveness and the ability to restructure and transform. In addition, many scholars have studied the indicators for measuring organizational capabilities, as shown in Table 3.

**Table 3:** Organizational capability measurement indicators.

Measure	Author
Investment capability, operation management, technological innovation and cultural management	Chandler (2019)
Organizational change capability, marketing capability	Wu Junji (2023)
Governance and leadership capabilities, internal operations and management, strategic relationships, resource development	Hart (2021)
R&D capability, production capability, marketing capability, technological innovation	Li Yangsheng (2022)
Network relationship capability and strategic capability	Sun jiaqi (2024)

##### 2.2.2.5 Organizational performance

Work performance refers to the quantity, quality, efficiency, and profitability of an organization’s task completion within a certain period. That is, the degree of achievement of organizational goals. The organizational resource advantages and capability advantages will ultimately be reflected in the performance level (Cameron, 1978).

Regarding the measurement of job performance, the setting of its dimensions varies from different disciplinary research perspectives. Cameron (1978) believed that job performance should include two dimensions: work effectiveness and work efficiency, and this view has been widely recognized. In his book “Human Resource Management”, French (1994) used two dimensions, organizational effectiveness and organizational efficiency, to measure job performance. Beck and Wilson (2000) believed that three dimensions—organizational efficiency, organizational effectiveness, and organizational development—can be used to measure job performance. The discussion on the definition and measurement dimensions of job performance has not yet reached a consistent conclusion (Wu Kunji, 2003). Venkatraman and Ramanujam (1986) believed that organizational performance should be measured using financial performance, operational performance, and overall performance. In addition, many scholars have studied the indicators for measuring work performance, as shown in Table 4.

**Table 4:** Metrics for measuring work performance.

Measure	Author
Net profit margin, sales profit margin, return on investment, own public and social image	Wang Hui et al. (2016)
Growth indicators, profitability indicators, market share	Lumpkin (1996)
Long-term performance, short-term performance	Lin Yiping (2021)
Finance, customers, business processes, learning and innovation	Wang Bingcheng (2024)

## 3 Research design and model

### 3.1 Research design

First, aiming at the initially identified research questions, combined with the current development status of sports associations, supplement and revise the research framework, continuously improve the structure of the thesis, and sort out the ideas of the thesis. Then, design the questionnaire for this study, conduct a pre-survey among the sports associations of Xinjiang University (Churchill, 1979), and perform data analysis after obtaining the data. Revise the questionnaire based on the data analysis results of the pre-survey and expert opinions, and conduct expert validity testing on the revised questionnaire.

Then, distribute and collect the formal questionnaires. The data of this thesis mainly comes from the questionnaire of “A Questionnaire Survey on the Impact of Network Relationship Strength on the Work Development of College Sports Associations”, with the survey objects being members of sports associations in undergraduate colleges in Urumqi.

This paper uses AMOS 24.0 and SPSS 24.0 statistical software, combined with research hypotheses and research models, to conduct data processing and related analysis on valid questionnaires, including descriptive statistical analysis, reliability testing, structural equation and general linear regression analysis, so as to test the theoretical hypotheses and research framework and obtain the statistical results of this study. After obtaining the data analysis results of the questionnaire survey, conduct interviews with experts and presidents of associations based on the results of the questionnaire survey.

Finally, combined with the results of the questionnaire survey and the interviews with experts and presidents of associations, analysed the results. At the same time, point out the deficiencies of this study and the prospects for subsequent research.

**Figure 1:**
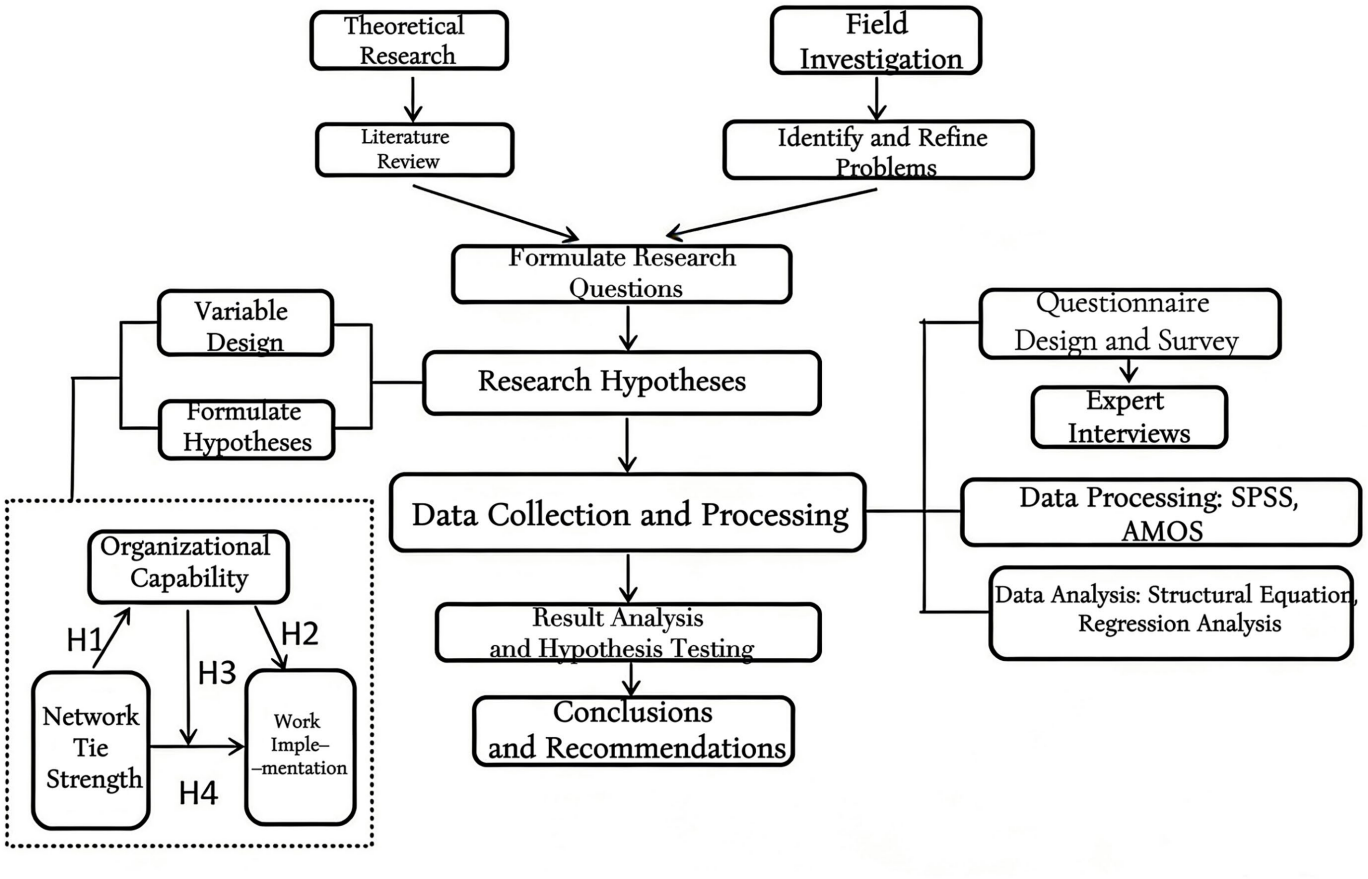
Research path.

The path of this study is shown in Figure 1.

This article puts forward the following hypothesis:

*H1*: There is a direct and significant positive correlation between the network relationship strength of university sports associations and work performance.

*H2*: The strength of the network relationships of college sports associations has a significant positive impact on their organizational capabilities.

*H3*: The organizational capacity of university sports associations has a direct and significant positive correlation impact on work performance.

*H4*: The organizational capacity of college sports associations plays an intermediary role between the strength of network relationships and the implementation of work.

### 3.2 Research model

#### 3.2.1 Statistical analysis of data

In accordance with the research questions of this paper, this paper will adopt methods such as descriptive statistical analysis, reliability test, correlation analysis, structural equation and regression analysis to test the proposed theoretical hypotheses (Koberg et al., 2003).

This study mainly uses AMOS 24.0 and SPSS 24.0 in statistical analysis software as the main data analysis tools. This study mainly uses AMOS 24.0 and SPSS 24.0 from statistical analysis software as the main data analysis tools.

AMOS is the abbreviation of Analysis of Moment Structures. It is a statistical software that uses structural equations to explore the relationships between variables (Escribano et al., 2009). It can analyze multiple variables simultaneously, perform structural equation modeling, and then conveniently and quickly create models to test the mutual influence and causes between multiple variables. Certain statistical software is used to process complex theoretical models, and the theoretical models are evaluated based on the degree of consistency between the models and the data, thereby confirming or falsifying the hypotheses proposed in this study. Moreover, SEM allows correlations between dependent variables because such correlations do not affect the analysis of the overall model path, thus having significant advantages over general linear statistics.

SPSS is the abbreviation of Statistical Product and Service Solutions. It is widely used in data mining, statistical analysis operations, predictive analysis, and decision support tasks, and is a statistical analysis software frequently used in research fields such as natural sciences, social sciences, and management. It has advantages in terms of ease of use, automatic statistical plotting, in-depth data analysis, and comprehensive functions. The specific order of data statistical analysis is as follows:

Step1: All the questionnaires recovered from the “Questionnaire Survey on the Impact of Network Relationship Strength on the Work of College Sports Associations” were coded and entered in accordance with the specific requirements of SPSS software to form the original database relied on in this study.
Step2: The data in the original database was filtered according to the specific requirements of this study, such as the region where the school is located, and the data that did not meet the research requirements was excluded, thus forming an effective database for this study.
Step3: Use SPSS to conduct reliability analysis, validity analysis, and descriptive statistical analysis on valid questionnaires.
Step4: Enter valid data into AMOS for structural equation modeling, and then use SPSS for regression analysis to conduct a robustness test on the mediating variables.
Step5: Classify valid data according to basic questions and establish conditional models, respectively.

#### 3.2.2 Pearson coefficient correlation analysis

Correlation analysis usually adopts Pearson coefficient correlation analysis for data analysis. In this paper, the reliability and factor analysis coefficients of each variable all reach an acceptable range, indicating that the setting of the question items in this theoretical model is reasonable and feasible (Gulati and Gargiulo, 1999). This study mainly involves three variables, and the mean score of each variable is greater than 3 points, which shows that the undergraduate college sports club members who participated in the questionnaire survey basically agree with the overall situation of network relationship strength, organizational ability, and work performance. The correlation coefficients between each pair of variables all reach a significant level significantly higher than 0.5 at the 0.01 level within the confidence interval (two-tailed), that is, there is a significant positive correlation between the three variables. Table 5 shows the correlation between each factor of network relationship strength, organizational ability, and work performance.

**Table 5:** Correlation analysis table between variable.

Variables	Significance	Strength of online relationships	Work performance
Strength of online relationships	Significance of Pearson correlation (two-tailed)	0.679	1.00
Organizational capability	Significance of Pearson correlation (two-tailed)	0.709	1.00
Work performance	Significance of Pearson correlation (two-tailed)	0.660	1.00

#### 3.2.3 Research model

**Figure 2:**
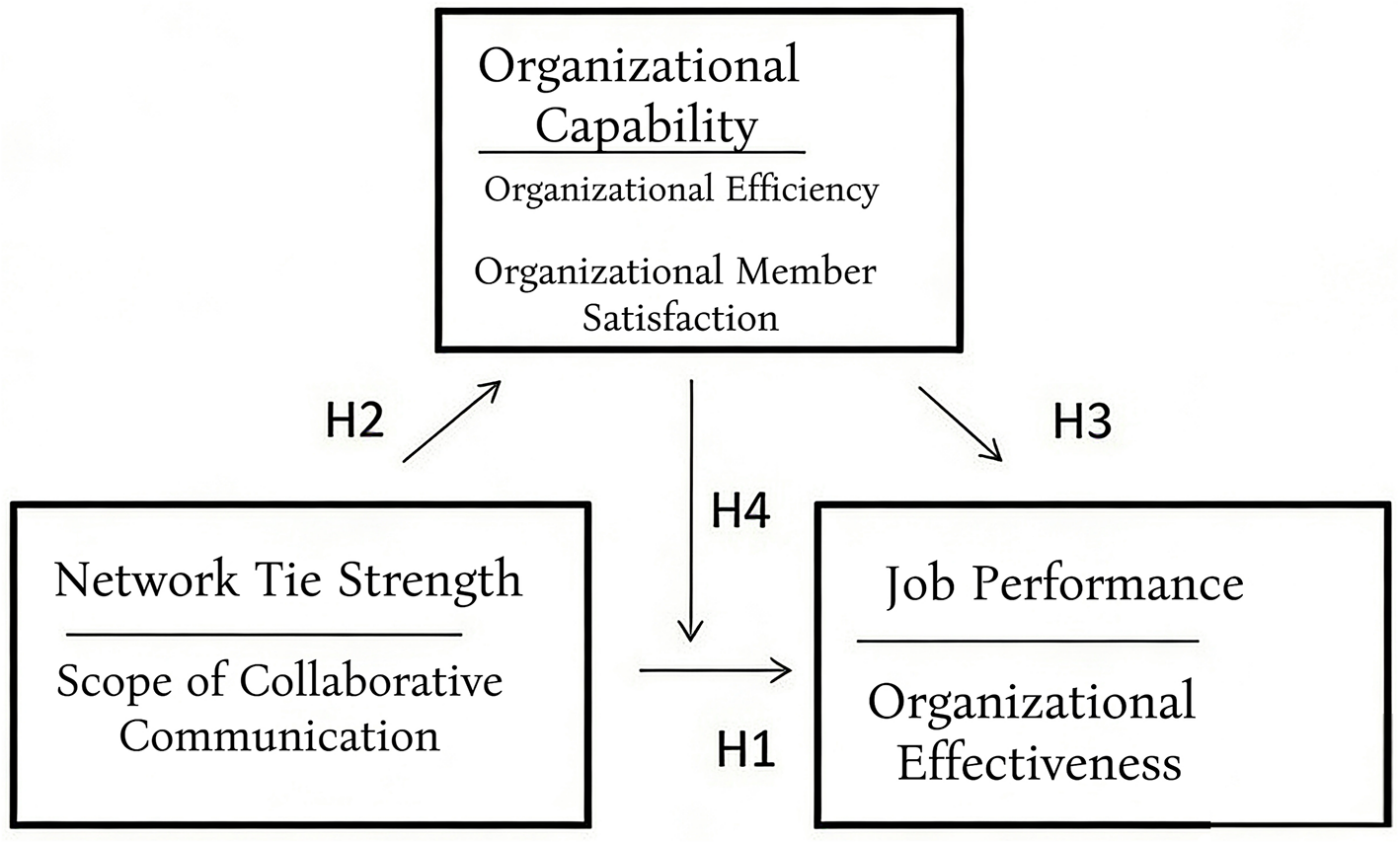
Research model.

This study takes the strength of network relationships as the main theoretical basis, combines the current development status of sports associations, and proposes the main research framework among the strength of network relationships, organizational capabilities, and work performance based on the previous literature review (Hart, 1995), as shown in Figure 2.

This study considers three variables—network relationship strength, organizational capacity, and work performance of university sports associations—within an integrated theoretical model. The fitting indices of this model are shown in the table below: *χ*^2^ = 39.202, df = 34, RMSEA = 0.011; CFI = 1.000, IFI = 1.000, both of which are greater than 0.90, reaching the ideal range. NFI = 0.998, RFI = 0.995, both of which are greater than 0.90, reaching the ideal range. The standardized chi-square value (*χ*^2^/df) = 1.153.

The mutual influence coefficients between each pair of network relationship strength, organizational capacity, and work performance of university sports associations are 0.494, 0.755, and 0.351, respectively (*p* = 0.000), reaching a significant level. A comprehensive analysis and judgment of each index show that the overall fitness of the data model is within an acceptable range, and the model has a good fit.

The role of the organizational capacity of university sports associations between network relationship strength and work performance is as high as 0.265. When the organizational capacity of university sports associations is included in the analysis of the relationship between network relationship strength and work performance, the influence of network relationship strength on work performance decreases significantly from the original 0.900 to 0.494. This is because organizational capacity plays a regulating role between the two, thus functioning as a partial mediator. Based on the analysis results of the data model, it can be concluded that the organizational capacity of college sports associations plays an obvious partial mediating role between the strength of network relationships and work performance, and Hypothesis 4 can be supported.

**Figure 3:**
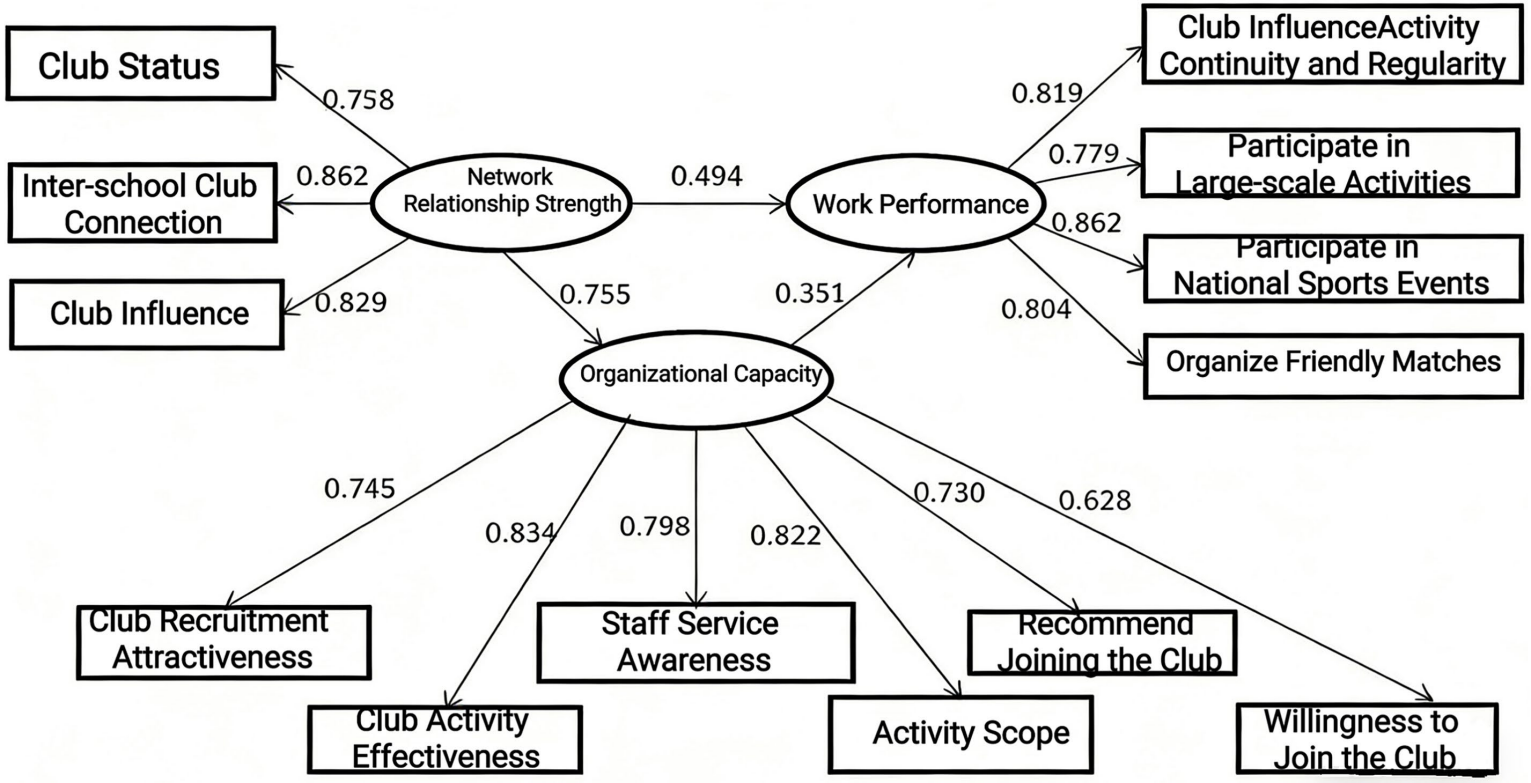
The overall relationship mode.

From this, we can derive the overall relationship model of this study based on the three variables (as shown in Figure 3).

## 4 Results

### 4.1 Descriptive statistics and reliability and validity analysis sections

All measurement scales in this study were adapted from mature international scales and revised according to the characteristics of university sports associations in China. Confirmatory factor analysis (CFA) was used to test the construct validity. The factor loadings of all items were between 0.72 and 0.89. The average variance extracted (AVE) values were 0.62, 0.58 and 0.60, and the composite reliability (CR) values were 0.87, 0.84 and 0.86 for network tie strength, organizational ability and organizational performance, respectively. All indicators meet the acceptable criteria in social science research.

The CFA model fit indices were *χ*^2^/df = 2.12, CFI = 0.97, TLI = 0.96, RMSEA = 0.048. The structural model fit was *χ*^2^/df = 2.35, CFI = 0.96, TLI = 0.95, RMSEA = 0.05. The mediating effect was tested using the Bootstrap method with 5,000 resamples, and the 95% confidence interval did not include 0, indicating a significant mediating effect.

### 4.2 Summary of hypothesis testing results

This study empirically verified four proposed research hypotheses, and all hypotheses were supported with statistically significant results: (1) The network tie strength of university sports associations has a significant positive impact on work performance (path coefficient = 0.90, *p* = 0.000); (2) Network tie strength has a significant positive impact on organizational capability (path coefficient = 0.755, *p* = 0.000); (3) Organizational capability has a significant positive impact on work performance (path coefficient = 0.351, *p* = 0.000); (4) Organizational capability plays a partial mediating role between network tie strength and work performance (mediating effect value = 0.265), and the direct effect of network tie strength on work performance decreases from 0.900 to 0.494 after the mediating variable is included in the model (Beck and Wilson, 2000).

The path coefficient from network tie strength to organizational ability is *β* = 0.52, which is relatively high. This is reasonable because college sports associations are typical resource-dependent non-profit organizations, whose organizational construction highly relies on external network resources. This result is consistent with similar studies on campus organizations.

### 4.3 Research results based on gender heterogeneity

To further explore whether the mutual influence relationships among the network relationship strength, organizational capacity, and work performance of college sports associations are affected by specific conditions (Levin and Cross, 2004), such as five conditions including students’ gender, school type, student status, exercise habits, and participation in sports associations (basic questions in this questionnaire), this paper classifies the original questionnaire data according to gender options and establishes the following models to clarify how the relationships among the network relationship strength, organizational capacity, and work performance of college sports associations change under the influence of the above gender conditions.

The original questionnaire data were classified by gender (1,103 males, 1,143 females) to explore the heterogeneous influence of network tie strength and organizational capability on work performance, and binary linear regression models were established (Y(JXA) = B + WL X1 + ZZ X2). The results showed that: (1) For male members, *R*^2^ = 0.517, both variables had significant positive impacts on work performance, and the impact of organizational capability was greater than that of network tie strength; (2) For female members, *R*^2^ = 0.577, both variables also had significant positive impacts on work performance, and the impact of network tie strength was greater than that of organizational capability.

In-depth interview results further verified the conclusions of the questionnaire survey: strong internal ties among core members improved the task execution efficiency of sports associations and reduced internal conflicts, while weak external ties with external organizations enhanced the association’s influence and resource acquisition capacity. In addition, institutional support was identified as a key contextual factor: sufficient institutional support (e.g., venues and funding) could facilitate the external cooperation of associations, while insufficient support would restrict their normal operational development.

### 4.4 Result analysis and discussion

a) In today’s era where collaborative exchanges are increasingly prevalent (Lowik et al., 2012), network relationships have become one of the important factors promoting the development of sports associations. The strength of relationships among members in the network relationships of college sports associations is an important condition for whether the associations can improve work performance. Therefore, based on previous studies, this paper puts forward Hypothesis H1: The strength of network relationships of college sports associations has a significant positive impact on work performance.

This study examined the relationship between the strength of network relationships and work performance of college sports associations through empirical research (Mitchell, 1974), explored the internal mechanism of the impact of relationship strength on work performance, and verified the establishment of Hypothesis H1 in the pairwise variable model (path coefficient is 0.90). That is, the more frequent the communication and cooperation among members in the network relationship, the more it can promote the improvement of the work performance of the association. Therefore, this study concludes that the strength of network relationships of college sports associations has a decisive direct impact on work performance, and strong relationships are conducive to promoting the improvement of work performance of college sports associations. The experts interviewed agreed with the results of our study, pointing out that strengthening exchanges with sports associations of other schools can accelerate the development of the association. The higher the frequency of exchanges, the more collisions between associations, which is more conducive to the development of the association.

The research results of this study are closely related to the current development status of sports associations, and the sample of this study—sports associations in undergraduate colleges and universities in Urumqi—has representative significance. For sports associations to achieve continuous development, they must continuously improve their work performance to enable the associations to play their due role. As mentioned earlier, it is particularly important for university sports associations to maintain certain communication and cooperation with other network members. By frequently contacting and communicating with other associations in the same school, associations in other schools, etc., sports associations can fully strengthen knowledge sharing among associations, create a good cooperative atmosphere, learn from each other’s experience in association organization, avoid risks in association management, and ultimately improve the work performance of the associations (Nelson and Winter, 1982). Therefore, the stronger the network relationship strength of university sports associations, the more it can promote the improvement of their work performance.

b) As mentioned earlier, in the network relationships of university sports associations, mutual communication and cooperation among members are conducive to achieving knowledge sharing and experience transfer between associations. Through cooperative communication, associations can identify their own shortcomings, learn excellent experiences from other associations, and promote their own development by organizing and absorbing these experiences within the association, which is the source of motivation for the development of associations. Therefore, this paper puts forward three hypotheses regarding the organizational capabilities of associations (Nonaka, 1994; Prahalad and Hamel, 1990). Hypothesis H2: The strength of network relationships of university sports associations has a significant positive impact on their organizational capabilities; Hypothesis H3: The organizational capabilities of university sports associations have a significant positive impact on their work performance. Hypothesis H4: The organizational capabilities of university sports associations play a significant mediating role between the strength of network relationships and work performance. That is, the work performance of university sports associations is indirectly affected by the strength of network relationships through the mediating variable of organizational capabilities. If the organizational capabilities of university sports associations match the strength of network relationships, the effect of the strength of network relationships on work performance will be further enhanced.

Through the analysis of sample data, it can be found that in both the pairwise variable models (with path coefficients of 0.75 and 0.76) and the overall theoretical model (with path coefficients of 0.755 and 0.351), the relationship between the network strength and organizational capabilities of university sports associations and the relationship between the organizational capabilities and work performance of university sports associations are such that Hypothesis H2 is supported, i.e., the stronger the relationship between members in the network of university sports associations, the stronger the organizational capabilities of the associations; at the same time, Hypothesis H3 is also supported, i.e., the stronger the organizational capabilities of university sports associations, the more they can promote the improvement of new work performance of the associations.

In the relationship between the strength of network relationships and organizational capabilities of university sports associations, organizational capabilities serve as the dependent variable; in the relationship between the organizational capabilities and work performance of university sports associations, organizational capabilities serve as the independent variable. From this, the specific influence path can be derived as follows: strength of network relationships of university sports associations → organizational capabilities → work performance. It can be seen that organizational capabilities play the role of an intermediate variable between the strength of network relationships and work performance of university sports associations.

The research results of this study are closely related to the current development status of sports associations in Urumqi, and the sample has representative significance for the development of university sports associations in Northwest China. However, due to the limitation of the research sample scope, the conclusions of this study should be cautiously generalized to other regions of China or foreign universities, and their applicability needs to be further verified by follow-up cross-regional research.

Combined with the verification of the previous hypotheses, the direct influence relationships between various variables are established. After adding organizational capabilities to the overall model, the influence coefficient of the strength of network relationships decreases from 0.900 to 0.494, indicating that organizational capabilities play a partial mediating role and are a key factor affecting the work performance of university sports associations. That is, the strength of network relationships of university sports associations can directly have a significant impact on work performance, and at the same time, it can also have an indirect impact on work performance through the mediating variable of organizational capabilities. This is an important finding of this paper and also a deepening and expansion of previous research results.

### 4.5 In-depth interview results

The in-depth interview results further verify the conclusions of the questionnaire survey. Most advisor teachers and core members of associations pointed out that strong ties among core members are the basis for maintaining the daily operation of the association, which can improve the efficiency of task execution and reduce internal conflicts. For example, a basketball association advisor teacher said: “The core members of the association have a good relationship, and they can quickly organize training and competitions, which ensures the stability of the association’s operations.” In addition, many core members mentioned that weak ties with external organizations are crucial for the development of the association. For example, a martial arts association core member said: “We have established cooperative relations with a local martial arts club, which not only provides us with professional coaches, but also helps us organize joint performances, which has greatly improved the influence of the association.”

Regarding the moderating role of institutional support, some interviewees pointed out that universities with sufficient institutional support can provide more venues and funding for associations, making it easier for associations to carry out external cooperation; on the contrary, universities with insufficient institutional support may restrict the development of associations. For example, a track and field association advisor teacher said: “The university does not provide enough funding, so we have to spend a lot of time looking for sponsorships, which affects the normal operation of the association.”

## 5 Discussion

### 5.1 Research conclusions and recommendations

a) The members of sports associations in undergraduate colleges and universities in Urumqi who participated in the questionnaire survey showed basically consistent recognition of the overall level of network tie strength, organizational capability and work performance, with no significant differences. The research conclusions based on this sample can reflect the development characteristics of university sports associations in Northwest China, but the generalization to other regions needs to be combined with the actual local development of university sports associations and further verified by empirical research. Combined with recent international studies (Jackson et al., 2023; Park and Kim, 2024), the positive effect of network tie strength is consistent with global findings. However, the mediating role is stronger in this study, which may result from differences in campus sports management systems between China and Western countries.
b) The strength of network relationships has a significant positive correlation impact on the work performance of university sports associations. Organizational capability plays a partial mediating role between the strength of network relationships and work performance of university sports associations. That is, the strength of network relationships can better affect work performance through the continuous improvement of organizational capability.
c) At this stage, the work performance of sports associations is not strong, mainly because the organizational capacity of the personnel in the associations is insufficient, resulting in the failure to reasonably and appropriately utilize various resources related to sports associations.
d) There are different influence mechanisms for the improvement of work performance between boys and girls in sports associations. Boys should pay more attention to the role of organizational ability, while girls should attach greater importance to the strength of network relationships to better improve work performance.

### 5.2 The potential shortcomings

The research sample is limited to 8 universities in Urumqi, which represents the development characteristics of higher education in northwest China. This regional limitation restricts the generalizability of the findings to eastern and central regions with more developed campus sports systems. Future studies are suggested to expand the sample to cover eastern, central and western regions of China to improve the universality of the conclusions.

This study has three core limitations that need to be emphasized, which also provide clear directions for the in-depth development of future research: First, limited geographical scope. The research sample is only limited to undergraduate universities in Urumqi, Northwest China, and the conclusions can only reflect the development characteristics of university sports associations in this specific region. They cannot be directly generalized to other regions of China or foreign universities with different institutional backgrounds, cultural environments and sports development foundations. Second, cross-sectional research design. This study only collects cross-sectional data at a single time point, which cannot reveal the dynamic evolution process and long-term impact mechanism of network tie strength on the operational development of university sports associations, nor capture the causal changes and interactive relationships of variables over time. Third, reliance on perceived measures only. The research data of all variables are obtained through self-reported questionnaires of sports association members, which are all subjective perceived measurement results. The study lacks objective secondary data (e.g., actual activity scale of associations, official operational performance evaluation data, statistics of resource acquisition volume) for cross-validation, which may lead to a certain deviation between the research results and the actual operational situation of university sports associations.

In addition, future research can further expand the research perspective by exploring the influence of network tie strength on the operational development of sports associations with different types and scales, so as to further improve and enrich the theoretical model of this study.

## Data Availability

The original contributions presented in the study are included in the article/supplementary material, further inquiries can be directed to the corresponding author.

## References

[ref1] Anderson J. C. Gerbing D. W. (1988). Structural equation modeling in practice: a review and recommended two-step approach. Psychol. Bull. 103, 411–423. doi: 10.1037/0033-2909.103.3.411

[ref2] Barden J. Q. (2007). Disentangling the influences of leaders relational embeddedness on interorganizational exchange. Acad. Manag. J. 50, 1440–1461. doi: 10.5465/amj.2007.28225983

[ref3] Baron R. M. Kenny D. A. (1986). The moderator-mediator variable distinction in social psychological research: conceptual, strategic, and statistical considerations. J. Pers. Soc. Psychol. 51, 1173–1182. doi: 10.1037/0022-3514.51.6.1173, 3806354

[ref4] Beck K. Wilson C. (2000). Development of affective organizational commitment: a cross-sequential examination of change with tenure, journal of vocational behaviour. J Vocational Behav 56, 114–136. doi: 10.1006/jvbe.1999.1712

[ref5] Blumstein P. Kollock P. (1988). Personal relationships. Annu. Rev. Sociol. 14, 467–490. doi: 10.1146/annurev.so.14.080188.002343

[ref6] Burt R. S. (1992). The Network Structure of Social Capital. Cambridge, MA: Harvard University Press.

[ref7] Cameron K. (1978). Measuring organizational effectiveness institution of high educating. Admin. Sci. Q. 23, 604–629. doi: 10.2307/2392582

[ref8] Chandler D. (1999). Economies of Scale and Scope in Enterprises——The Driving Force of Industrial Capitalism. Beijing: China Social Sciences Press.

[ref9] Churchill G. A. (1979). A paradigm for developing better measure of marketing constructs. J. Mark. Res. 16, 64–73. doi: 10.1177/002224377901600110

[ref9004] Eisenhardt K. M. (2000). Paradoxes of speed. Calif. Manag. Rev. 42, 34–50.

[ref10] Escribano A. Fosfuri A. Tribó J. A. (2009). Managing external knowledge flows: the moderating role of absorptive capacity. Res. Policy 38, 96–105. doi: 10.1016/j.respol.2008.10.022

[ref9007] French W. L. (1994). Human Resource Management. 6th Edn. Boston: Houghton Mifflin.

[ref9005] Gold A. H. Malhotra A. Segars A. H. (2001). Knowledge management: an organizational capabilities perspective. J. Manag. Inf. Syst. 18, 185–214.

[ref9003] Granovetter M. S. (1973). The strength of weak ties. Am. J. Sociol. 78, 1360–1380.

[ref11] Gulati R. Gargiulo M. (1999). Where do interorganizational networks. Strateg. Manage. J. 104, 1439–1493. doi: 10.1086/210179

[ref12] Hart O. (1995). Corporate governance: some theory and implications. Econ. J. 105, 678–689. doi: 10.2307/2235027

[ref9009] Jackson R. (2023). Sports organization network and operational performance. Sport Management Review 26, 512–528.

[ref13] Koberg C. Koberg D. Detienne K. (2003). An empirical test of environmental, organizational, and process factors affecting incremental and radical innovation. J. High Technol. Manag. Res. 14, 21–45. doi: 10.1016/S1047-8310(03)00003-8

[ref14] Levin D. Z. Cross R. (2004). The strength of weak ties you can trust: the mediating role of trust in effective knowledge transfer. Manag. Sci. 50, 1477–1490. doi: 10.1287/mnsc.1030.0136

[ref9012] Lin Y. P. (2021). Long-term vs. short-term organizational performance. J. Organ. Behav. 42, 789–806.

[ref15] Lowik S. Van Rossum D. Kraaijenbrink J. Groen A. (2012). Strong ties as sources of new knowledge: how small firms innovate through bridging capabilities. J. Small Bus. Manage. 50, 239–256. doi: 10.1111/j.1540-627x.2012.00352.x

[ref9013] Lumpkin G. T. Dess G. G. (1996). Clarifying the entrepreneurial orientation construct and linking it to performance. Acad. Manag. Rev. 21, 135–172.

[ref16] Mitchell J. C. (1974). Social networks. Annu. Rev. Anthropol. 3, 279–299. doi: 10.1146/annurev.an.03.100174.001431

[ref17] Nelson R. Winter S. (1982). An Evolutionary Theory of Economic Change. Cambridge: Harvard University Press.

[ref18] Nonaka I. (1994). A dynamic theory of organizational knowledge creation. Organ. Sci. 5, 514–537.

[ref9010] Park J. Kim S. (2024). The mediating role of organizational capability in sports association networks. Asian Sport Management Review 15, 89–105.

[ref19] Prahalad C. K. Hamel G. (1990). The core competence of the corporation. Harv. Bus. Rev. 68, 79–92.

[ref9006] Teece D. J. Pisano G. Shuen A. (1997). Dynamic capabilities and strategic management. Strateg. Manag. J. 18, 509–533.

[ref9002] Venkatraman N. Ramanujam V. (1986). Measurement of business performance in strategy research. Acad. Manag. Rev. 11, 801–814.

[ref9011] Wang B. C. (2024). Performance evaluation of university student organizations. Higher Education Forum 3, 67–70. (in Chinese)

[ref9001] Wang H. . (2016). Net profit margin, sales profit margin, return on investment, public and social image. J. Manag. Stud. 42, 1567–1589.

[ref9008] Wu K. J. (2003). Research on organizational performance evaluation of Chinese enterprises. Manage. World 10, 145–146. (in Chinese)

